# A promising RNA nanotechnology in clinical therapeutics: a future perspective narrative review

**DOI:** 10.2144/fsoa-2023-0067

**Published:** 2023-07-18

**Authors:** Mahmoud M Tolba, Abdul Jabbar, Sadia Afzal, Mohammed Mahmoud, Farheen Zulfiqar, Ingy El-Soudany, Salma Samir, Al-Hassan Soliman Wadan, Takwa E Ellakwa, Doha El-Sayed Ellakwa

**Affiliations:** 1 Pharmaceutical Division, Ministry of health & population, Faiyum, Egypt; 2 Department of Veterinary Medicine, Faculty of Veterinary Science, University of Veterinary & Animal Sciences, Lahore Punjab, Pakistan; 3 Department of Botany, Faculty of Life Science, Women University Multan; 4 Faculty of Pharmacy, Ain Shams University, Cario, Egypt; 5 Department of Food Science & Human Nutrition, University of Veterinary & Animal Sciences Lahore Punjab Pakistan; 6 Microbiology & Immunology Department, Faculty of Pharmacy, Pharos University in Alexandria, Alexandria, Egypt; 7 Genetics & Genetic engineering Department, Faculty of Agriculture, Benha University; 8 Faculty of Dentistry, Sinai University, Arish Branch, North Sinai, Egypt; 9 Physical Chemistry, Faculty of Pharmacy, Egyptian Russian University, Egypt; 10 Department of Biochemistry & Molecular Biology, Faculty of Pharmacy for Girls, Al-Azhar University, Cairo, Egypt; 11 Department of Biochemistry, Faculty of Pharmacy, Sinai University, Kantra Branch, Ismailia, Egypt

**Keywords:** endosomal escape, endosomes, nanoparticles, physicochemical properties, ribose nucleic acid, RNA nanotechnology

## Abstract

Nanotechnology is the use of materials that have unique nanoscale properties. In recent years, nanotechnologies have shown promising results for human health, especially in cancer treatment. The self-assembly characteristic of RNA is a powerful bottom-up approach to the design and creation of nanostructures through interdisciplinary biological, chemical and physical techniques. The use of RNA nanotechnology in therapeutics is about to be realized. This review discusses different kinds of nano-based drug delivery systems and their characteristic features.

## Plain language summary

A branch of nanotechnology called RNA nanotechnology involves designing, studying, and utilizing synthetic structures based on RNA. This review discusses different kinds of nano-based drug delivery systems and their characteristic features. It aims to provide an overview of nanoparticles as a delivery system for gene therapy to treat diseases such as cancer. In order to enhance nanoparticle efficacy, these systems should be designed with this in mind in order to develop and test delivery systems rationally and scientifically.

There is a broad spectrum of nanoparticles (NPs) with dimensions ranging from 1 nm up to 100 nm that have emerged as an amazing class of materials. The physical and chemical properties of nanoparticles have made them useful in a wide range of scientific and technological applications. Advanced nanotechnology is often considered a universal technology due to its potential to impact almost all aspects of society and industries significantly. Its impact is significant in medicine, healthcare, biotechnology, and information technology [1]. Nanoparticles have been used for therapeutic drugs and gene delivery in medicine, developing treatment systems for various disorders [2]. This article serves as a guide for selecting the most suitable RNA nanotechnology for therapeutics and can help identify novel RNA nanotechnology with the desired features.

## Classification of nanoparticles based on physicochemical properties

### Lipid-based NPs

**Figure 1.**
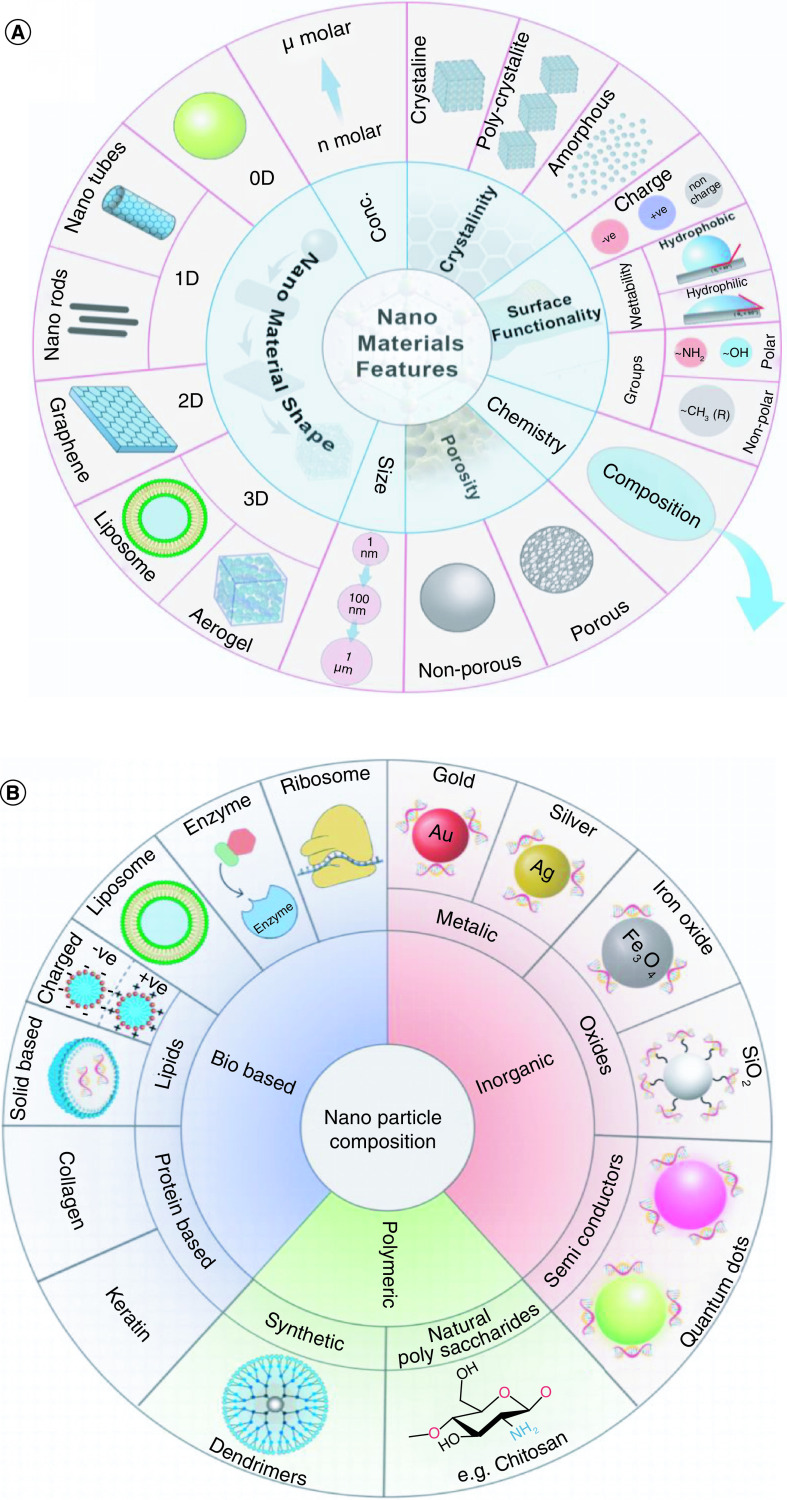
Types of nanomaterials and composites considered in this review and different types of nanoparticles divided into organic, polymeric, bio-based and inorganic categories. **(A)** Features and types of nanomaterials. **(B)** Classifications of nanoparticles based on composition.

Many NPs had been developed based on fatty components. NPs formed of solid fat and named solid lipid nanoparticles (SLNs) are fat-based colloidal mixture systems with a solid pattern at the body temperature. Lipid-based NPs, often known as lipid nanoparticles (LNPs), are a subclass of organic NPs. They consist of at least one lipid bilayer encircling at least one interior aqueous compartment, and are typically spherical platforms. As a delivery vehicle, lipid-based NPs have a number of benefits, such as easy formulation, self-assembly, biocompatibility, high bioavailability, capacity for large payloads, and a variety of physicochemical properties that can be tuned to influence biological features. LNPs, which differ from conventional liposomes in that they form micellar structures within the particle core and can have different morphologies depending on the formulation and synthesis conditions, are frequently utilised for the transport of nucleic acids. The most prevalent class of FDA-approved nanomedicines is lipid-based NPs [3]. The composition of SLNs is usually a reliable water-repellent centre (traditionally made of triglycerides) covered by phospholipids. Liposomes have also been utilized as lipid-based NPs for holding DNA. These NPs lead to DNA penetration into the cells owing to liposomal capability to merge with biological membranes [4]. Identical to liposomes, SLNs were used as carriers for many drugs to many organs, such as the lungs [5] as summarized in Figure 1 A & B & (Table 1).

**Table 1.** Composition, characteristics, and optimal trials of several NPs.

Type of NPs	Composition	Properties	Successful attempts/ treatments	Ref.
Lipid NPs	– Globe shaped manifold coated cavity – Consist of organic and inorganic lipid phosphate and steroids.	Capability to merge with biological layers	– Utilized to transmit FUSI to tumor cells of lungs. – Outcome; reduction in magnitude of cancerous cells.	[6]
Chitosan NPs	– Positively charged – Polysaccharide produced by basic de-acetylation of N-terminal of chitin.	– Has both polycation and poly anion characteristics which permits it to establish firm electrostatic interconnections with deoxyribose nucleic acid and anionic mucosal surfaces. – Have the capability of making firm complex compounds with deoxyribose nucleic acid as little as twenty-five hundred nanometer in magnitude. – Its proficiency in adhering is connected to its de-acetylation potential and molecular weight.	– Gastrointestinal tract inoculation of chitosan-based insulin genetic compounds to rats that have diabetes. – Outcome: rapid reduction in the amount of FBS of the rats inoculated with chitosan-DNA particles.	[7]
Dendrimers	A structure in which particles have equal size i.e., monodispersed – Regularly-structured mostly branched three-dimensional format. – Consist of an inner core, inner coatings of polymers, and an outer functional coating adhered to the outermost inner generations.	– High effectiveness in transmission (poly amidoamine dendrimers). – Has amine groups which work in deoxyribose nucleic acid linking and enhancing deoxyribose nucleic acid phagocytosis (pri amines), and assist the emission of deoxyribose nucleic acid into the cytoplasm by functioning as a proto-sponge in endosomes (ter amines).	– Utilized for long-lasting medication of neurological disease [Parkinson's] when mix with plasmid deoxyribose nucleic acid containing glial cell derived neurotrophic factor gene. – Outcome: An enhancement in the number of line crossings and inert time, and the dropping of dopaminergic neurons was decreased.	[8]
Gold NPs		– Inactivity. – Facility of working with thiol interconnections. – Plasmon reverberation.	– Stop the copying of T7 ribose nucleic acid polymerase utilizing NPs of gold functioning with trimethylammonium thiol. – Utilizing NPs of gold based (GNOME) laser transmission technology to enhance the transmission proficiency of Au NPs.	[9]

NP: Nanoparticle.

### Polymer-based nanoparticles

Many polymer-based NPs (PNPs) have been used to fabricate self-assembled polymer-based NPs as delivery systems. The use of nanotechnology in the medical profession is known as nanomedicine, and it involves the use of nanoparticles with sizes in the nanoscale range. As a field of study in science, drug delivery systems are becoming more and more significant. Polymers are increasingly being used more frequently, and controlled-release systems and drug-targeting systems offer an alternative to conventional delivery nanoparticles [10]. They can be categorized by being hydrophilic or hydrophobic, by their physical behaviors like pH sensitive polymers or thermosensitive polymers and many other types of classification. Examples of hydrophobic polymers, the polymers containing poly (propylene oxide), polystyrene and poly (lactide-co-glycoside). Examples of Hydrophilic polymers; those containing poly (ethylene oxide) segments in their structure and poly (ethylene glycol). Poly (acrylic acid) is an example of a pH-sensitive polymer, while polyphosphoesters are temperature-sensitive polymers. Some of these polymers are charged, and this charge is beneficial in the case of gene encapsulation. For example, positively charged polymers can interconnect with negatively charged genomic material because of the amino acid groups in their structures that adhere to the deoxyribonucleic acid of the gene. These polyplex deoxyribonucleic acid compounds are incorporated into cells through absorptive or receptor-mediated engulfing and eventually proceed toward lysosomes [11].

Another polymer type is the organic polymers or biopolymers. Living species generate those polymers and are made of proteins and polysaccharides that make them biocompatible and non-immunogenic [12]. Protein-based biopolymers are known for their high stability, water-binding capacity and good gelation and emulsification. These properties enable biopolymers to act as excellent biodegradable carrier [13]. The composition of protein-based biopolymers can be further controlled by encrypting their arrangement at the genomic level by utilizing genetic engineering technology. The most common natural protein-based biopolymers include collagen and elastin protein [14].

A very well-known polymer-based gene carrier is the Chitosan–DNA complex NPs. Cationic polymers like chitosan can manage the release of antibiotics, proteins, deoxyribose nucleic acid, vaccines or proteinaceous drugs. These abilities enabled cationic polymers to be used in the pharmaceutical industry and research. Cationic polymers have also been studied extensively as non-viral deoxyribose nucleic acid vectors for genetic transmission and medication. Chitosan can make firm electrostatic interconnections with deoxyribose nucleic acid and anionic mucosal surfaces. The adhering proficiency of chitosan depends on its de-acetylation degree and molecular mass. Some studies reported that some chitosan grades could be utilized for organic approaches such as immunization [15].

### Dendrimers

A type of radially symmetric, nanoscale molecules known as dendrimers have a distinct, homogenous, and monodisperse structure. They have an exterior shell, an inner shell, and a center that is normally symmetric. Several dendrimers are effective delivery or carrier systems for medicines and genes because they have intrinsic pharmacodynamic characteristics. However, some dendrimers' cytotoxicity prevents them from being used in medicine. Unlike most linear polymers, dendrimers can be synthesized with a degree of control that yields essentially monodisperse, globular macromolecules with a significant number of peripheral groups. Numerous uses for dendrimer prodrugs can be found in developing novel medications [16]. Dendrimers are similar to polymers in having repeated units. Still, they are different from polymers because they possess highly organized structures owing to their method of preparation that involves step-by-step production. Another difference is that dendrimers are characterized by three-dimensional design. Dendrimers can imitate the unimolecular micelles by having similar structures composed of a lipophilic internal core and hydrophilic surface shell. Dendrimers can be used in gene and drug delivery owing to their ability to enhance encapsulated materials' solubility, which will also enhance their bioavailability. Moreover, being large enables dendrimers to escape filtration by the kidneys. On the other hand, keeping the nanometric size range will lead to enhanced penetration and retention effect [8].

### Inorganic NPs

Inorganic NPs include metals, metal oxides, metal sulphides and semiconductors. Inorganic, non-metallic elements are combined to form microscopic particles called norganic nanoparticles, which are then heated, cooled, and then given certain properties. Nanostructured materials (NSMs) like zinc oxide (ZnO) and titanium dioxide (TiO_2_) as well as semiconductors like silicon and ceramics are examples of inorganic-based nanomaterials. Inorganic NPs are employed in a variety of applications including catalysis, water treatment, medicine, electronics, agriculture, chemical catalysis, the food industry, and many more because of their special qualities. Additionally, some metal NPs have special thermal, magnetic, and biological characteristics, making them valuable building blocks for nanodevices with a variety of physical, chemical, biological, medicinal, and pharmacological uses [17]. They are widely used in numerous applications as gene delivery vectors because of their optical and magnetic properties and high surface-to-volume ratio.

#### Metallic NPs

Metal NPs are colloidal metallic solids, e.g., silver and gold. These metal NPs are characterized by being neither deposited nor forming conglomeration [18]. Metal-only precursors are used to create metallic nanoparticles, which can be monometallic, bimetallic, or polymetallic. Metallic NPs have distinct physical and chemical features because of their small size and large surface area, including localized surface plasmon resonance characteristics, distinct optical and electrical properties, and distinct thermal, magnetic, and biological properties. The most researched metallic NPs are gold NPs which are employed in a variety of structures such nanospheres, nanorods, nanostars, nanoshells, and nanocages. Metallic NPs can be created to have a wide range of sizes, topologies, and geometries since they are accurately formed. Metallic NPs are used in a wide range of industries, including the food industry, electronics, agriculture, water treatment, medicine, and catalysis [19]. The process of metal NPs preparation involves using sodium citrate or sodium borohydride to reduce metal salts. Afterward, the handling with polymer-thiols curbing coating for sustaining. Rao *et al.*, 2007 suggested that Romans mastered using metal colloids for purposes including treating arthritis and dying glasswork and fabrics [20]. Nevertheless, it was M. Faraday who was the foremost person to observe that there are discrepancies in the optical properties between gold colloids and bulk gold [21]. Nowadays, the prime research centre is to realize the potential of gold NPs in gene therapy through calibrating the magnitude and structure and operating their surface [22], stimulation [23], noting and observing old deoxyribose nucleic acid [24], tumor imagination [25], together with many other practices.

##### Gold nanoparticles

Gold NPs are considered a promising delivery system because of their properties such as dormancy, ease of functioning with R-SH interconnections and plasmin reverberation. Such properties underwent a comprehensive analysis to investigate their clinical implementations [7]. It was reported in a study that the successful assessment of gold nanoparticles as nucleic acid vectors after being used to inhibit the copying of T7 ribonucleic acid polymerase utilizing NPs of gold functioning with trimethylammonium thiol [26]. Several studies were done on gold NPs to increase their transfection efficiency or control the selective release of nucleic acids. For instance, the imaging and physical characteristics of NPs of gold were used to mainly emit two different deoxyribose nucleic acids conjoined from two other packs of gold nanosized rods [27].

##### Metal oxides NPs

Superparamagnetic iron oxide NPs (SPIONs) are a group of ultra-fine, magnetic NPs, prepared with ferric oxide, including ferric [III] oxide (Fe_2_O_3_), ferrous [II] oxide (FeO), and magnetite ferrosol ferric oxide (Fe_3_O_4_) [28]. SPIONs with specific coating can be adhered to specific drugs, genetic material or immunoglobulins and guided toward particular locations for transmission [29]. To prevent their aggregation, these NPs are always surface-functionalized. Surface functioning utilizes three sorts of matter, synthetic compounds such as Silicon dioxide (SiO_2_), biological molecules such as fatty acids, and surface-active agents such as oleic acid (sodium salt) [30].

##### Semiconductor NPs & Quantum dots

Semiconductors incorporate NPs are tiny nanoscale crystals of magnitude ranging from 1 nm to hundred nanometers [31]. Mainly utilized NPs are quantum dots, which are characterized by being fluorescent, colloidal and wavelength-tunable. The size and shape of quantum dots can be controlled by adjusting both the duration and temperature of the synthesis process or the kind of binding molecule utilized [31]. Quantum dots comprise a center and an outer layer that shields the nanosized crystal from decomposition. Cadmium selenide and zinc sulphide are renowned quantum dots having a core and outer layer consisting of cadmium selenide (CdSe) core and outer layer of zinc sulphide (ZnS). After being synthesized, cadmium selenide and zinc sulphide can be dissolved through ligand interchange and then become bio functionating to provide several biological and therapeutical practices [32]. It was reported that functionalized quantum dots could make either covalent or electrostatic conjunction with nucleic acids [9].

## Benefits of ribose nucleic acid nano-applications for *In vivo* practices

Recently, several kinds of clinical ribose nucleic acid NPs have been produced and extensively investigated for their potential use in diverse disease management. *In vitro* gene silencing has been achieved with high efficiency and particularity using various interface techniques and ribonucleic acid molecules; however, *in vivo*, the proficient transmission of medicinal ribonucleic acids to particular cells still faces obstacles.

## Polyvalent transmission for producing harmonious effects

The polyvalent ribonucleic acid NPs represents a delivery system that is characterized by the elasticity for making polyvalent transmission machines that can transmit up to six different types of molecules to particular cells involving drugs, analytical molecules or therapeutics [6]. In this delivery method, single ribonucleic acid subunits with different contents can be prepared individually. Following construction, RNA subunits are mixed together in any desired combination. They were then collected into the concluding Qua complex compound [33]. The other subunits of ribonucleic acid NPs act as a supplying method for anti-cancer drugs to improve their efficacy, or even can be included in a combination therapy to overcome the drug resistance. One NP through a single administration, has the ability to combine the either therapeutic detection molecules or drugs, or both. Thus, improve both the therapeutic effect and the detection of therapeutics [33].

### Nanoscale magnitude for improved absorptive & reservation effects

Delivery of the drug to a diseased tissue through NP technology mainly depends on the nanometer range particle size of the formed system [34]. According to several studies, the optimal size for a NP ranges from 10–100 nm [34]. This range is successful because NPs are both sufficiently huge to neglect secretion through the urine and adequately small to adhere to the receptors of the cell surface and then penetrate the cell through receptor-based cell eating [34]. Several studies reported that the pharmacokinetics, pharmacodynamics, and biological distribution of Small interfering RNA [siRNA] NPs delivery systems are improved with reduced toxicity [35].

## Obstacles & suggested solutions faced in ribonucleic acid with nano technique

**Figure 2.**
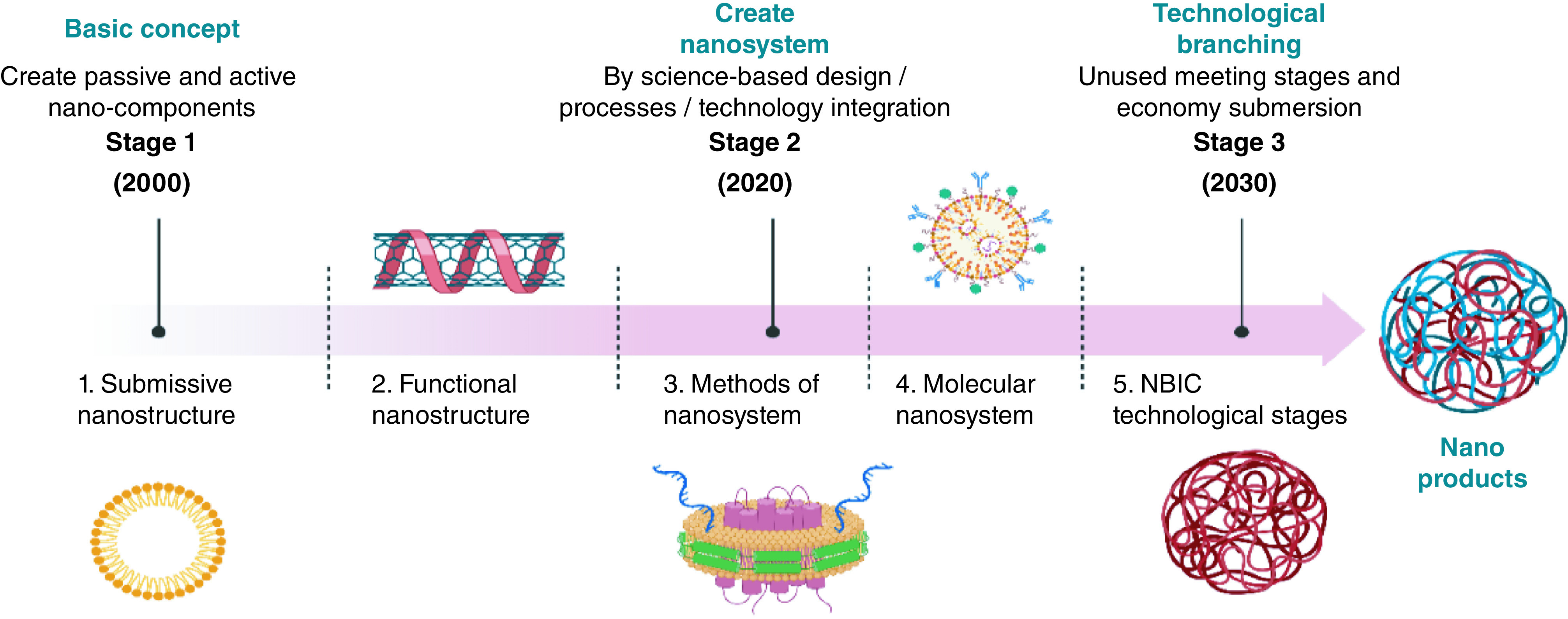
The generation of nanotechnology in different stages with future perspective.

Some answers and prospects are provided herein for the diversity of problems involving synthetic and thermal behaviours uncertainty, short biological half-life, low productivity and expensive manufacturing techniques, possible adverse effects, and a breakout of endosomes (Figure 2).

The chemical stability of RNA represents one of the main issues to be considered to ensure the long-term utilization of RNA NPs in therapeutics. Innate ribonucleic acid has maximum vulnerability toward deterioration by ribonuclease enzymes with more fluctuation in the body or serum. Numerous strategies have been examined over the last few years to reinforce the steadiness of RNA, counting synthetic alterations of the bases (e.g., 5-Bromouracil and 5-I-Uracil); adjustments of the phosphate interconnections [e.g., thiophosphate, borane salts BH3 consist of a phosphate group]; modification of the C at two prime [for example 2 prime fluorine, two prime methoxy group or two prime amino groups] [36], capped deoxyribonucleic acids and ribonucleic acids, and their relevant derived forms; nucleic acids polymers having carbamates units [37] or bolted nucleic acids with a connection at different locations [two prime,-four prime, one prime-three prime] [37]; and restriction at the 3 prime end [38]. All these strategies demonstrated their adequacy toward expanding ribonuclease opposition. Nevertheless, the real obstacle to succeeding synthetic alteration is represented by the change in both the overlapping characteristics and the bio functions of the ribonucleic acid molecule. Therefore, there is an escalating need to create a process that improves opposition to deterioration by Ribonuclease [RNase] without altering the specific properties of the construction, self-assemblage or bio functions of ribonucleic acid NPs. It was observed that the 2′-F has a negligible negative impact on overlapping assemblage and working [39,40]. Moreover, RNase degradation *in vivo* was not a priority due to fine-tuning that makes a difference in looking for an area that can be adjusted with negligible inconvenient impact. Besides, it has appeared that vulnerable sites where RNA degradation in serum occurs are frequently fine-tuned by mutation or alteration [41].

### Endosome trapping

**Figure 3.**
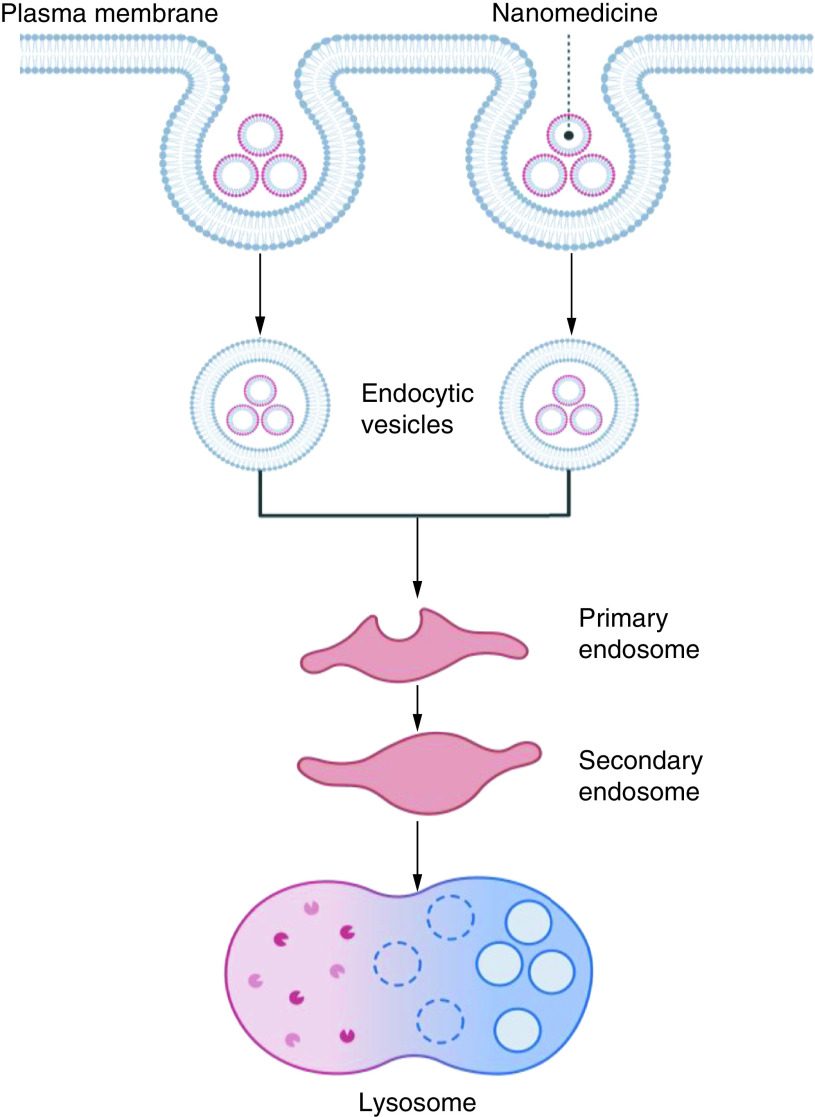
Showing mechanisms of endosomal escape.

The debasement of medicinal compounds within the passage of endocytosis is a major issue in DNA or protein delivery. Endosomal escape is the most significant obstacle in the ligand-based cellular capture for particular transmission of small interfering ribonucleic acid or clinical ribonucleic acid NPs Figure 3. The NPs are caught within the endosomes inside the cells following receptor-mediated endocytosis. Hence, siRNA is kept from being processed by the dicer apparatus and cannot disassemble a mainly aimed genetic material [42].

Oligomerization properties of promoter-associated RNAs (pRNAs) can move the conveyance of clinical compounds in forming a network with endosome debating operators, which can disturb endosomes and consequently intercede endosome elude of therapeutic molecules. Imperfect or psoralene-unfunctional adenovirus components have significant endosomatic action [43]. Polymeric endosome-disturbing genetic transmission carriers such as poly (amino esters) [44] or poly (lactic-co-glycolic acid) (poly PLGA) [45] have been detailed as engineered proteinaceous bonds that recreate the membrane-combining locale of the Hema glutenin of the flu virus. And these peptides can be effectively utilized in gene conveyance framework to encourage break out from endosomes [46]. Polymers facilitating a few synthetic elements have been detailed to improve the disruption of the membrane-bound vesicle endosome. The endosome-escaping reagents are often consolidated into the RNA NPs utilizing the characteristics of having more than one valency of ribonucleic acid NPs. One or two fractional parts of the ribonucleic acid compound are often changed to carry constituents that encourage disturbance of membrane-bound vesicles (endosomes) for the discharge of the conveyed clinical molecules from the endosomes. Besides, one fractional part of the transferable ribonucleic acid compound (Dimeric, Trimeric, and Hexamer) is often changed to incorporate a ribonucleic acid aptamer which behaves as a binder for receptors of the surface of the cell and actuates take up by receptor-based cell eating upon adhering. The opposite fractional parts of the ribonucleic acid compound are often utilized to contain a small interfering ribonucleic acid, a catalytically active ribonucleic acid molecule, micro ribonucleic acid, a riboswitch, or a synthetic sedate.

There are several strategies to help endosomal elude. These strategies incorporate the utilization of chemically prepared polymers to make small interfering ribonucleic acid or polymer polyplex, the linking of small interfering ribonucleic acid with fats to make NPS of fat or the small interfering ribonucleic acid affiliation with the proteinaceous material that pierce cells (cell-penetrating peptides) or synthetic material that disturb membrane-bound vesicles (endosomes). The arrangement of small interfering ribonucleic acid or polymer polyplex for discharge from endosomes and genetic quieting effectiveness has design considerations that have been, as of late, checked [47,48].

Regarding acid-splittable binders, the positively charged end of the polymer's multiplexes gained protonation beneath the impact of pH <7 of the membrane-bound vesicle (endosome). Consequently, the membrane-bound vesicle (endosome) is destabilized, employing the proton sponge impact. In addition, the acid hydrolysis followed by the loss of the cationic branches comes about within the decreased interconnections between small interfering ribonucleic acid and polymer, which in turn, leads to the discharge of the small interfering ribonucleic acid for endoribonuclease dicer or helicase processing [49].

Cell penetrating peptides [CPPs] speaks to other technique for small interfering ribonucleic acid transmission and disordering of endosomes. Since CPPs ordinarily convey small interfering ribonucleic acid into the cells in a similar pathway as small interfering ribonucleic acids or polymer polyplex and NPs containing fat. After interaction with negatively charged small interfering ribonucleic acid, positively charged proteinaceous material carries siRNA beside them and enters the cell through endocytosis. Amphipathic peptides procured the most centre for nucleic acid delivery because they are brief and contain a large number of histidine and leucine buildups. [50] They all appeared to effectively deliver small interfering ribonucleic acid for the genetic material of enzyme luciferase to mammal cells. This productivity was comparable to common lipid-incorporated transmission agents such as the agent lipofectamine (cationic liposome). Despite this unclear process of endosomal disturbance by amphipathic proteinaceous material, it is mostly that pH <7 of the endosome guides toward the protein makeup of the amino acid (His) proliferation, which liberates the proteinaceous material from small interfering ribonucleic acid they were containing and permits them to disturb membrane-bound vesicles (endosomes) through protonation wipe impact. After collecting weak bases in the endosome, the proton sponge effect takes place. The proton sponge neutralizes the endosome's lumen and expands the endosome's osmolarity [51]. Subsequently, the membrane-bound vesicles (endosomes) develop and lose their capacity to hold their substance, driving the discharge of siRNA into the cytosol.

A later report examined the pH reliance of endosome elude employing an economically accessible peptide (having properties of both polar and non-polar components) called endo-porter that has been utilized to provide various contents of nucleus material (DNA and RNA) to the cells of mammals [52]. Endo-porter requires the acidification of the endosome. In other words, Endo-porter makes a secondary confirmation, such as α-helix at the pH between 5.0 and 6.0, while it does not occur at physiological pH. The precise method for endosome disruption by the α-helical structure is still unknown. However, it may be an outcome of the interconnection between the membrane of vesicles (endosomes) and the alpha-helix driving the arrangement of a massive opening in the membrane-bound vesicle (endosomes) or distortion of its coating.

Despite their various sorts, there is still a significant challenge related to using NPs capable of endosomal disturbance in therapeutics [53]. The need for specificity incredibly restrained the convenience of the industrially prepared polymer NPs, fat-containing NPs, and amphiphilic peptides *in vivo* [54]. Focusing on elements such as ribonucleic acid aptamers or receptor-aiming ligands, such as vitamin B12, is considered one of the preferences of RNA NPs. Though, endosomal elude is still considered an impediment. In case of combining into one particle, a mainly aiming ribonucleic acid NP can be made to evade the membrane-bound vesicles (endosomes) [55]. This nanoparticle can clear the way toward a modern and practical shape of RNA therapeutics.

## Conclusion

Clearly, nanotechnology has improved healthcare by facilitating advances in the biotechnology, pharmaceutical, and medicinal industries. Cancer targeting has been improved with RNA nanotechnology, but efforts are required to boost delivery efficiency and specificity to individual cancer cells. In addition, combining RNA with other chemical polymers to augment endosomal escape has faced the obstacles of undefined structure, stoichiometry, and nanoparticle accumulation in normal organs.

## Future perspective

Nanoparticles are a vital delivery system in the field of gene therapy for managing diverse diseases like cancer. The self-assembly characteristic of RNA can be considered as a powerful bottom-up approach for the design and creation of nanostructures through the interdisciplinary of biological, chemical, and physical techniques. Many research groups, including ours, are working to solve the endosome escape issues. Once these challenges are overcome, the field of RNA nanotechnology and therapeutics will move into clinical reality.

**Executive summary**

**Classification of nanoparticles (NPs) based on physiochemical properties**

- Nanoparticles (1–100 nm) are capable of treating cancer due to their specific advantages, such as biocompatibility, low toxicities, excellent stability, increased permeability and retention, and precise targeting.
- Pharmaceutics has been utilizing nanoparticles for decades to reduce side effects and toxicity.

**Benefits of RNA-applications for *in vivo* practices**

- Establishing an effective, non-pathogenic and practical nanosized gadget for different clinical delivery *in vivo* is necessary.
- Therapeutic RNAs have been developed, and their potential for treating diseases is just beginning to be explored.

**Obstacles & suggested solutions faced in RNA with nano technique**

- A major hurdle in pursuing RNA as a therapeutic remains specific targeting and endosome escape.

**Conclusion**

- Pharmaceutics has been utilizing nanoparticles for decades to reduce side effects and toxicity.
- The use of nanoparticle delivery systems could improve the treatment of many diseases.
- Field of RNA nanotechnology and therapeutics will move into clinical reality once solving the endosome escapes issues.

**Future perspective**

- A rapid spread of nanotechnology in medicine, specifically drug delivery, is expected.
- For clinically approved RNA nanoparticles to be optimized, the immunostimulation by these nanomaterials will need to be overcome.
